# New Generation IVF – What does it mean?

**Published:** 2015-12-28

**Authors:** Wim Ombelet

**Affiliations:** Genk Institute for Fertility Technology, ZOL Hospitals, Schiepse Bos 6, 3600 Genk, Belgium.

**Keywords:** Adjuvants, affordable, genetics, cost-effective, IVF, new technology

## Abstract

What will be the future of IVF taking into account the growing attention of health care providers: will providing the best success-rate per trial by using more and more innovative techniques become our only goal or should we also focus on a strategy to reduce costs wherever possible. A go-together of both strategies seems to be the best option to convince governments and health care providers to further support the use of assisted reproductive technologies. To provide cost-effective, high quality and affordable IVF should be our main concern not only from an economical but also from an ethical point of view

Recent discoveries in reproductive biology and medicine were anything short of remarkable. Although we all know that the development of new techniques is the way to progress, proper assessment should be a prerequisite before general use.

According to the review of Datta et al. (2015) in this issue, many new technologies and adjuvant therapies have been advocated in order to improve the success rate of IVF since many years; most of them are being practiced on a large scale despite little justifiable evidence to support their use.

Endometrial injury, advanced sperm selection procedures, preimplantation genetic screening, time-lapse embryo monitoring, assisted hatching, different drugs and immune therapy to support the luteal phase and to increase the implantation rate etc.; all of these adjuvant therapies seem to be very attractive not only for clinicians and biologists working in the field of human reproduction, but also for the media and consequently for many infertile couples desperately searching for a child. One cannot deny that all these new interventions and additional drugs make IVF treatment more expensive with questionable actual advantage.

The findings described in pilot-studies such as the one of Mendez Lozano et al. (2015) in this issue on tadalafil have to be verified in a randomized controlled trial before recommending the use of this drug in an IVF programme.

When talking about the future of assisted reproduction it seems that two trends become visible. According to many, the future of assisted reproduction is very clear: ICSI for all, genetic screening on all embryos before transfer, no value of andrologic work-up (only the semen counts), no future for intra-uterine inseminations as a first-line therapy (why should we use this technique if IVF is very successful), limited use of endoscopic surgery etc. They believe that “New generation IVF” means 100% success per transfer and the transfer of an embryo without genetic screening has to be regarded as a serious mistake. In their view optimal air quality is the only prerequisite for IVF laboratories, whatever it costs. They support the idea of timelapse embryo monitoring in all IVF laboratories, preimplantation genetic screening of all embryos and the routine use of the endometrial receptivity array (ERA), an array based on the expression of 238 genes capable of diagnosing a functionally receptive endometrium and allowing the personalization of the optimal day for embryo transfer (Simon et al., 2015). Achieving the highest success rate is their most important goal; the price to pay for the patients and the society is undoubtedlynot their first worry.

Another group of experts focus much more on cost-effectiveness, patient-friendly IVF, prevention of complications such as the ovarian hyperstimulation syndrome (OHSS) and multiple pregnancies and the development of methods aiming to increase access to IVF and related techniques. They support and investigate the use of mild ovarian stimulation protocols not only to decrease the costs but also to decrease the burden for the patients (Fauser et al., 2010; Ferraretti et al., 2015).

More and more infertility experts believe that the implementation of OHSS-free clinics and the road to a freeze-all strategy is a timely one to travel. They claim that freezing all embryos will minimize many of the costly and potentially complicating procedures engendered in fresh cycle transfers, as we currently know them. This also means that more research is needed to develop optimal cryostorage approaches and restoring as best we can the properties of gametes and embryos.

New methods to decrease the costs of the IVF laboratory have proven to be successful, with a huge reduction in costs (Van Blerkom et al., 2014). The worldwide implementation of such simplified methods, especially in the resource-poor countries, will surely increase global access to infertility care.

At present the price to pay for IVF/ICSI, whether paid by the patients themselves, the government or the insurance companies, varies between 4000 and 15000 Euro per cycle. If we can’t make these methods less expensive, IVF treatment will be limited to a small part of the world population. I cannot imagine that this was Bob Edwards’ dream when he developed this technique.

As mentioned in the paper of Datta et al. (2015), a lot of new technologies and drugs are used without any scientific evidence. Most of these new methods and adjuvant therapies are very expensive and used worldwide without proven benefit.

Global access to infertility care should be the major goal for all players in the field of reproductive health. High quality IVF can be done at affordable prices by using less expensive ovarian stimulation protocols combined with simplified culture techniques. This doesn’t mean that new technologies are not important anymore, on the contrary.

The importance of the genetic revolution and the expected benefits for the infertile couple cannot be underestimated. On the other hand, we have to be very careful to implement new techniques that are too expensive or not supported by good scientific evidence, including cost-effectiveness.

As completion of the fourth decade of ARTs fast approaches, I sincerely hope that “New generation IVF” will be a go-together of both trends, aiming to achieve optimal chances to pregnancy and healthy offspring at affordable costs, with special attention to the needs of the infertile patients themselves.
